# Tissue Type-Specific Expression of the dsRNA-Binding Protein 76 and Genome-Wide Elucidation of Its Target mRNAs

**DOI:** 10.1371/journal.pone.0011710

**Published:** 2010-07-23

**Authors:** Valentina Neplioueva, Elena Y. Dobrikova, Neelanjan Mukherjee, Jack D. Keene, Matthias Gromeier

**Affiliations:** 1 Division of Neurosurgery, Department of Surgery, Duke University Medical Center, Durham, North Carolina, United States of America; 2 Department of Molecular Genetics and Microbiology, Duke University Medical Center, Durham, North Carolina, United States of America; City of Hope National Medical Center, United States of America

## Abstract

**Background:**

RNA-binding proteins accompany all steps in the life of mRNAs and provide dynamic gene regulatory functions for rapid adjustment to changing extra- or intracellular conditions. The association of RNA-binding proteins with their targets is regulated through changing subcellular distribution, post-translational modification or association with other proteins.

**Methodology:**

We demonstrate that the dsRNA binding protein 76 (DRBP76), synonymous with nuclear factor 90, displays inherently distinct tissue type-specific subcellular distribution in the normal human central nervous system and in malignant brain tumors of glial origin. Altered subcellular localization and isoform distribution in malignant glioma indicate that tumor-specific changes in DRBP76-related gene products and their regulatory functions may contribute to the formation and/or maintenance of these tumors. To identify endogenous mRNA targets of DRBP76, we performed RNA-immunoprecipitation and genome-wide microarray analyses in HEK293 cells, and identified specific classes of transcripts encoding critical functions in cellular metabolism.

**Significance:**

Our data suggest that physiologic DRBP76 expression, isoform distribution and subcellular localization are profoundly altered upon malignant transformation. Thus, the functional role of DRBP76 in co- or post-transcriptional gene regulation may contribute to the neoplastic phenotype.

## Introduction

All mRNA functions, starting with biogenesis, co-transcriptional processing, nuclear export, subcellular distribution, translation and ending with decay, involve association with RNA-binding proteins forming discrete ribonucleo-protein (RNP) complexes [1], [2]. Therefore, through binding to functionally related transcripts, individual RNA-binding proteins have the potential for affecting gene regulation by coordinating control over mRNA function [3].

**Figure 1:**
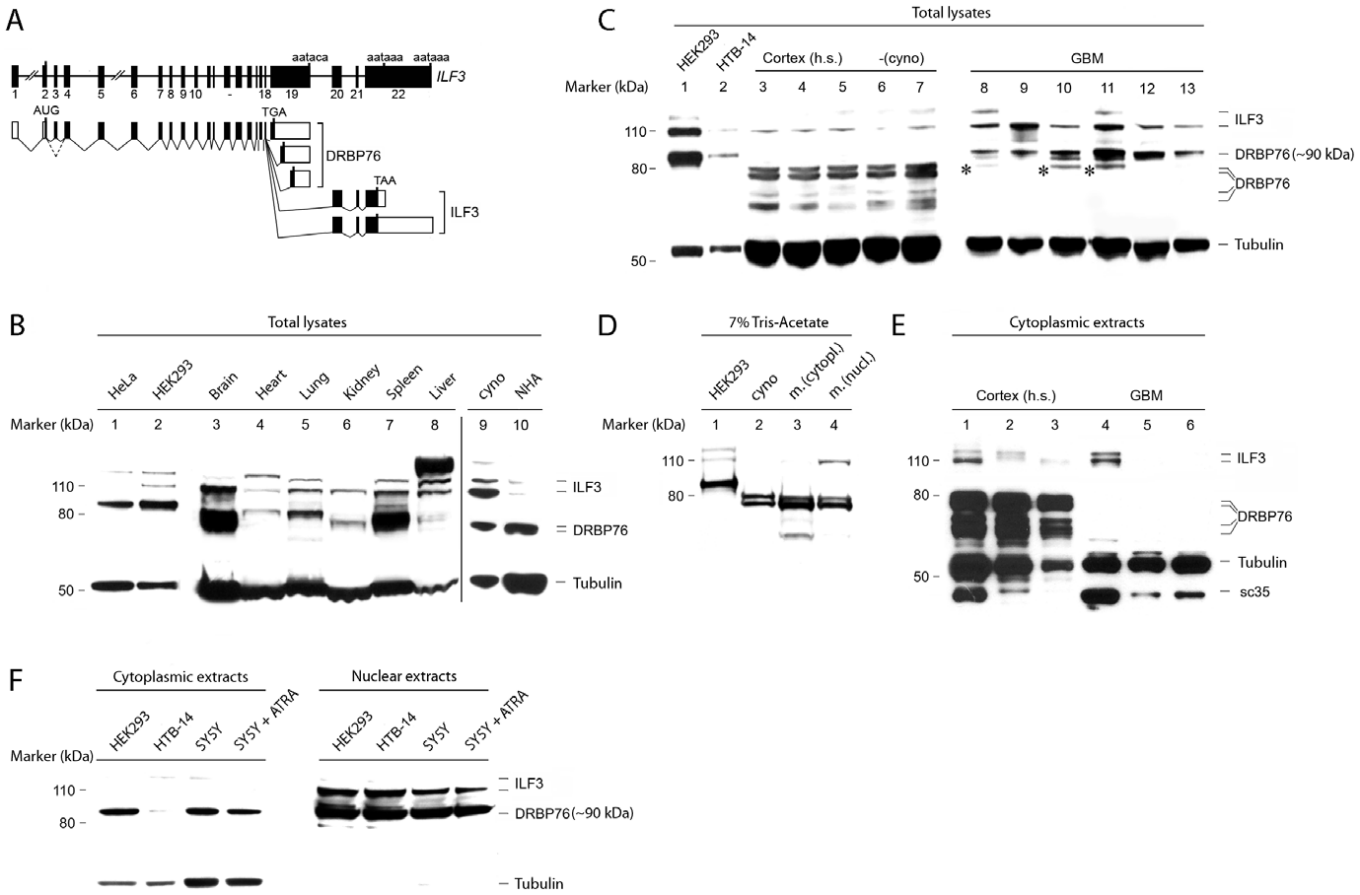
NFAR protein isoform- and subcellular distribution in normal and neoplastic tissues. **A**. Intron-exon organization of the *ILF3* gene [25], [33]. Exons are indicated by black boxes (top); alternative splicing yields diverse transcripts encoding DRBP76/ILF3 proteins with distinct C-termini (bottom; coding sequences are indicated by solid boxes). Hatched lines indicate proposed alternative splicing of exon 3 [25]. **B**. DRBP76 immunoblot of HeLa or HEK293 cell lysates (lanes 1–2) or non-fractionated murine tissue extracts of the indicated origin (lanes 3–8). (Lanes 9–10) DRBP76 immunoblot of normal *Cynomologus* brain (cyno) and NHA cell lysate. **C**. DRBP76 immunoblots of HEK293 and HTB-14 glioma cell lysates (lanes 1, 2) and total extracts prepared from normal cortex (3, 6 = frontal; 4, 7 = temporal; 5 = parietal) of human (h.s.) or simian (cyno) brain or human GBM tissue samples (8–13). Asterisks indicate histopathologically confirmed contamination with normal CNS. **D**. NFAR protein immunoblot analyzed with 7% tris-acetate gel electrophoresis. Simian (cyno) and fractionated cytoplasmic and nuclear murine (m.) brain extracts were studied in parallel with HEK293 lysates. Protein bands are labeled in (E). **E**. Evaluation of NFAR proteins in fractionated, cytoplasmic extracts from human normal brain and GBM tissues. Immunoblot filters were deliberately over-exposed to reveal all DRBP76-immunoreactive material. sc35 is a nuclear spliceosome component. **F**. DRBP76 immunoblot from fractionated extracts of the indicated cell lines. SH-SY5Y (SY5Y) cells were untreated or treated to induce a neuronal phenotype with ATRA.

The *interleukin enhancer binding factor 3* (*ILF3*) gene encodes a variety of related proteins in cultured cells with apparent sizes of 110 kDa (termed ILF3 [4] or nuclear factor associated with dsRNA-2 (NFAR-2) [5]) and 90 kDa (termed nuclear factor (NF) 90 [6], [7], DRBP76 [8] or NFAR-1 [5]). Diverse NFAR proteins originate from alternative splicing in the 3′ portion of *ILF3* transcripts (Fig. 1A). For clarity, we will refer to the 110 kDa and 90 kDa proteins as ILF3 and DRBP76, respectively. Both proteins are known to associate with nuclear factor 45 (NF45), forming the nuclear factor of activated T-cells (NFAT; [9]). NFAR proteins contain nucleic acid binding motifs [8] and were originally identified because of their association with the *interleukin-2* promoter [7]. They have been implicated in transcriptional regulation, although their precise role in this process remains poorly defined [10]. In addition, DRBP76 contains two double-stranded (ds) RNA binding motifs [11]. Complementing its involvement in transcriptional regulation, many lines of evidence point to a role for DRBP76 in post-transcriptional gene regulation due to its RNA-binding capacity.

DRBP76 has been suggested to interact with a variety of cellular or viral RNAs, both in the nucleus and in the cytoplasm (reviewed in [12]). It was identified in screens for AU-rich element (ARE)-binding proteins [13], [14], but its various proposed target mRNAs generally do not share apparent recognition signals. Association with DRBP76 in the cytoplasm has been shown to stabilize target mRNAs [14], [15] and to influence their translation [14], [16]–[20]. However, based on disparate observations in diverse empirical systems, no uniform role in post-transcriptional gene control has been assigned to NFAR proteins.

It is well established that DRBP76 shuttles in and out of the nucleus [21] and, thus, obvious that any function in post-transcriptional gene regulation relates to its subcellular distribution. Based on studies in immortalized cultured cells of diverse origin it has been suggested that DRBP76 is predominantly nuclear and may translocate to the cytoplasm when prompted by certain stimuli, e.g. post-translational modification during mitosis [21]. It has been suggested that the subcellular distribution of DRBP76 is in part determined by protein:RNA interactions in either the nuclear or cytoplasmic compartments [21]. However, there is emerging evidence that subcellular DRBP76 distribution may vary inherently in a cell type-specific manner. This is evident from studies with HEK293 cells, showing a predominantly cytoplasmic distribution of the protein [11], [12], [18], [19]. This raises the possibility that DRBP76 assumes cell type- or organ-specific roles in gene regulation.

We report here that DRBP76 exhibits strikingly distinct isoform expression and subcellular distribution in the normal human central nervous system (CNS) and in glial CNS malignancy, reminiscent of earlier observations in cultured cells [18]. Abundant cytoplasmic DRBP76 in normal CNS contrasts with exclusive nuclear distribution in patients' tumors. These observations suggest that DRBP76's involvement in gene regulation may vary in a tissue type-specific manner. To define the pool of mRNAs associating with DRBP76, we carried out RNA co-immunoprecipitation (IP)/micro-array (RIP-Chip) assays [22], [23] in HEK293 cells. Our analyses revealed statistically significant associations of DRBP76 with distinct classes of transcripts key to the regulation of metabolism, DNA synthesis and cell proliferation.

## Results

### 1. Tissue type-specific distribution of DRBP76 isoforms

We tested DRBP76 isoform distribution in an array of adult mouse tissues (the anti-DRBP76 antibody used recognizes primate/murine proteins alike; Fig. 1B, D). For orientation, a proposed intron-exon organization of the *ILF3* gene and the origin of diverse NFAR proteins are shown (Fig. 1A). In general agreement with previously published work [24], we detected several proteins of a predicted size range of ∼75–80 kDa (presumably DRBP76) and ∼110–120 kDa (presumably ILF3) in all organs tested (Fig. 1B). However, our analyses suggest that DRBP76 is substantially more abundant in the nervous system and spleen compared to other organ systems. This is particularly obvious when considering the ILF3:DRBP76 expression differential in individual organs (e.g., brain vs. liver). We carefully normalized tissue lysates for total protein content and controlled our assay by evaluating the levels of several marker proteins, including tubulin (Fig. 1B) and eukaryotic initiation factor 4A (not shown). Moreover, our analyses suggested that DRBP76 proteins, but not ILF3, differ in apparent size in normal adult mouse tissues and in human transformed cell lines (Fig. 1B).

To corroborate these findings, we further evaluated DRBP76 proteins in a major expression site, the CNS. We tested normal human brain tissues from various brain regions obtained at autopsy (Fig. 1C, lanes 3–5). Due to concerns with possible post-mortem degradation of the sample, we included brain from *Cynomolgus* macaques, tissues that were fresh frozen immediately upon euthanasia and dissection of the brain (Fig. 1C, lanes 6, 7). Paralleling earlier studies with representative cell lines of spontaneous tumors of astrocytic lineage [18], we also evaluated DRBP76 distribution in tissues from patients diagnosed with primary CNS tumors, i.e. glioblastoma multiforme (GBM). Under the specific electrophoresis conditions employed we distinguished at least two major DRBP76 isoforms of ∼75 to 80 kDa in normal brain, while a single form of ∼90 kDa predominates in HEK293 and HTB-14 glioma cells. A similar DRBP76 doublet in immunoblots of mouse brain lysates has been observed previously [25], but that study did not show comparison with cell lines.

Interestingly, all primary CNS tumors displayed NFAR protein isoform distribution similar to transformed cell lines (Fig. 1C, lanes 8–13). In some tumors a band reminiscent of the major ∼80 kDa sized protein in normal CNS was detected (Fig. 1C, asterisks), but these samples contained contaminating normal brain, a frequent occurrence in such samples that was indicated in the neuropathologist's report. These analyses suggest that physiologic DRBP76 isoform distribution is altered upon malignant transformation, at least in the CNS. The appearance of NFAR proteins in primary CNS tumors is comparable to transformed cells regardless of their tissue origin (e.g. HeLa cells; Fig. 1B, lane 1), suggesting that altered DRBP76 isoform distribution may occur in other neoplasms. Since GBM is of glial origin, normal primate brain may not be an appropriate non-malignant control tissue. Therefore, we evaluated DRBP76 isoform distribution in normal human astrocytes (NHA; Fig. 1B, lane 10). These cells recapitulated the DRBP76 isoform distribution detected in normal brain (compare Fig. 1B; lane 9). Therefore, altered DRBP76 isoform expression in GBM is a true variation from the non-malignant state and does not represent a glia-specific phenomenon. Also, since primary explant human cells retain the DRBP76 isoform pattern of their organ of origin, DRBP76 isoform patterns in HeLa or HEK293 cells may reflect their transformed state.

### 2. Tissue type-specific subcellular localization of DRBP76

DRBP76 contains a functional nuclear localization signal and its location is predominantly nuclear in many cultured cell lines [10]. It has been suggested that certain physiologic conditions, e.g. T-cell activation [15] or mitosis [21], may trigger cytoplasmic localization of the protein, possibly via post-translational modification or modulation of RNA binding [21]. We showed previously that ∼90 kDa DRBP76 is enriched in the cytoplasm of cultured cells of neuronal origin in the absence of known re-localization triggers when compared to non-neuronal cells, e.g. malignant glioma [18].

Extensive heterogeneity of NFAR proteins in mammalian brain has been observed before [25] and may be due to splice variation [25] and post-translational modifications, e.g., arginine methylation [26], lysine acetylation [27] or phosphorylation [8]. To better resolve anti-DRBP76 immunoreactive material in the 75–90 kDa size range and to begin assessing subcellular localization of NFAR proteins in the normal CNS, we tested fractionated (cytoplasmic vs. nuclear) tissue lysates by immunoblotting after gel electrophoresis in 7% Tris-acetate gels (Fig. 1D). The characteristic doublet of bands at ∼80 kDa occurs in mouse and *Cynomolgus* macaque brain and is clearly distinguishable from a band with larger molecular size in HEK293 cells (Fig. 1D, lane 1). Exclusive detection of the ∼110 kDa band and less abundant ∼80 kDa material in nuclear extracts (Fig. 1D, lane 4) suggests that the former may represent ILF3 and the latter to occur in the cytoplasm.

To evaluate cytoplasmic DRBP76 in the normal human brain and in GBM, we tested fractionated cytosolic extracts by immunoblot (Fig. 1E). Surprisingly, while highly abundant in normal brain (lanes 1–3), cytosolic DRBP76 of ∼75–80 kDa was absent from immunoblots of fractionated cytoplasmic human GBM samples (lanes 4–6) (Fig. 1E). We included detection of sc35, a nuclear spliceosome component, to evaluate the possibility of nuclear contamination. These tests revealed readily detectable nuclear contaminants in samples 1 (brain) and 4 (GBM), which correlated with the presence of ILF3 in both samples (Fig. 1E, lanes 1, 4). Judging from the band intensity in correlation with sc35, ILF3 was roughly equally abundant in normal brain and in GBM tissues. Our analyses indicate that the ∼75–80 kDa DRBP76 protein is abundant in normal human brain, and it is absent, or below detection level, in tissues from primary CNS tumors.

To test whether different subcellular distribution of DRBP76 occurs in transformed cells of diverse origin, we conducted DRBP76 immunoblot studies in fractionated cell extracts from HEK293 neuroblasts, SH-SY5Y neuroblastoma cells and HTB-14 glioma cells. Similar to previous reports, the bulk of DRBP76 was present in nuclear fractions (Fig. 1F). However, there was abundant cytoplasmic DRBP76 in the neuronally derived cell lines, but hardly any cytoplasmic protein in HTB-14 cells (Fig. 1F). Induction of a neuronal phenotype with all-trans retinoic acid (ATRA) treatment of SH-SY5Y did not change the isoform pattern or subcellular distribution of DRBP76 (Fig. 1F). These findings suggest that transformed cell lines of neuronal origin acquire the ‘malignant’ isoform pattern associated with primary CNS tumors, but retain at least some cytoplasmic DRBP76 characteristic of normal CNS tissues.

**Figure 2:**
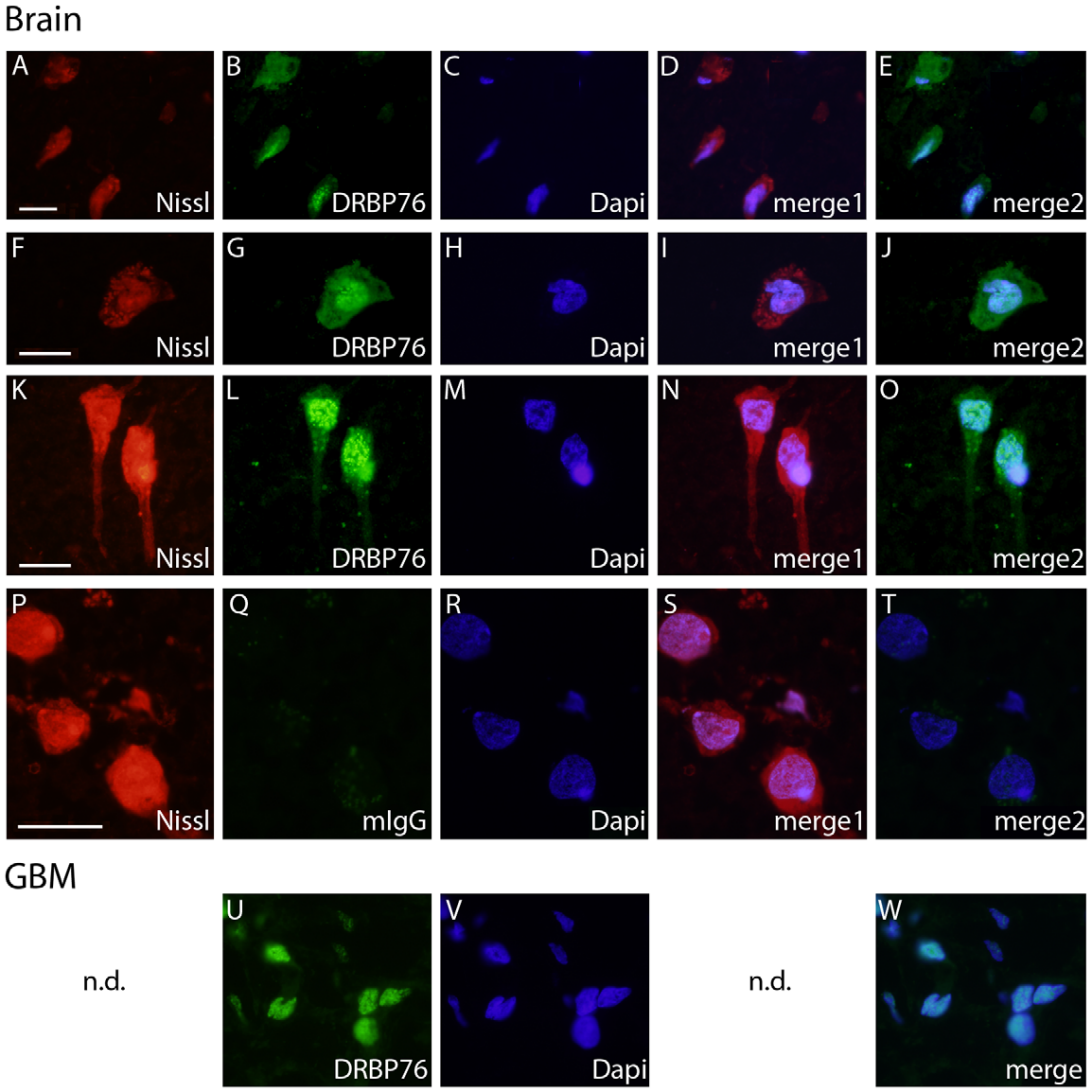
Immunofluorescence analyses of NFAR proteins in the human brain and in GBM. **A.–O**. NFAR proteins in human brain. Cryostat sections of normal brain were treated with the indicated detection reagents. Merge 1 (Nissl:DRBP76 antibody) and 2 (Dapi:DRBP76 antibody) suggest cytoplasmic NFAR proteins in neurons. **P.–T**. Control immunofluorescence with isotype-matched non-specific mouse IgG. Merge 1 (Nissl:mIgG) and 2 (Dapi:mIgG) suggest the absence of non-specific staining. **U.–W**. NFAR proteins in GBM. The merged image (Dapi:DRBP76 antibody) suggests exclusive nuclear NFAR proteins in GBM cells. Bars = 50 µm.

To corroborate the different subcellular localization of DRBP76 proteins in normal human brain and in GBM, we conducted immunofluorescence studies in both tissues. We assayed histopathologically confirmed normal brain (Fig. 2A–T) and GBM free of contaminating normal CNS tissue (Fig. 2U–W). Nissl substance, abundant in neuronal cells, was readily detected by NeuroTrace fluorescent stain in sections of normal human brain (Fig. 2A, F, K, P). Immunostaining of the same sections with anti-DRBP76 (Fig. 2B, G, L) revealed the presence of NFAR proteins in both nucleus and cytoplasm of large pyramidal neurons (Fig. 2D–E, I–J, N–O). Non-specific staining was assessed using isotype-matched mouse IgG (Fig. 2P–T). In GBM sections, staining with anti-DRBP76 occurred only in areas with DAPI signal (Fig. 2U–W), indicating exclusively nuclear localization of NFAR proteins. Thus, immunofluorescence analyses suggested that NFAR proteins are present in the cytoplasm of normal neurons (Fig. 2A–O), but not in GBM (Fig. 2U–W).

### 3. Immunoprecipitation of DRBP76 and associated mRNPs

**Figure 3:**
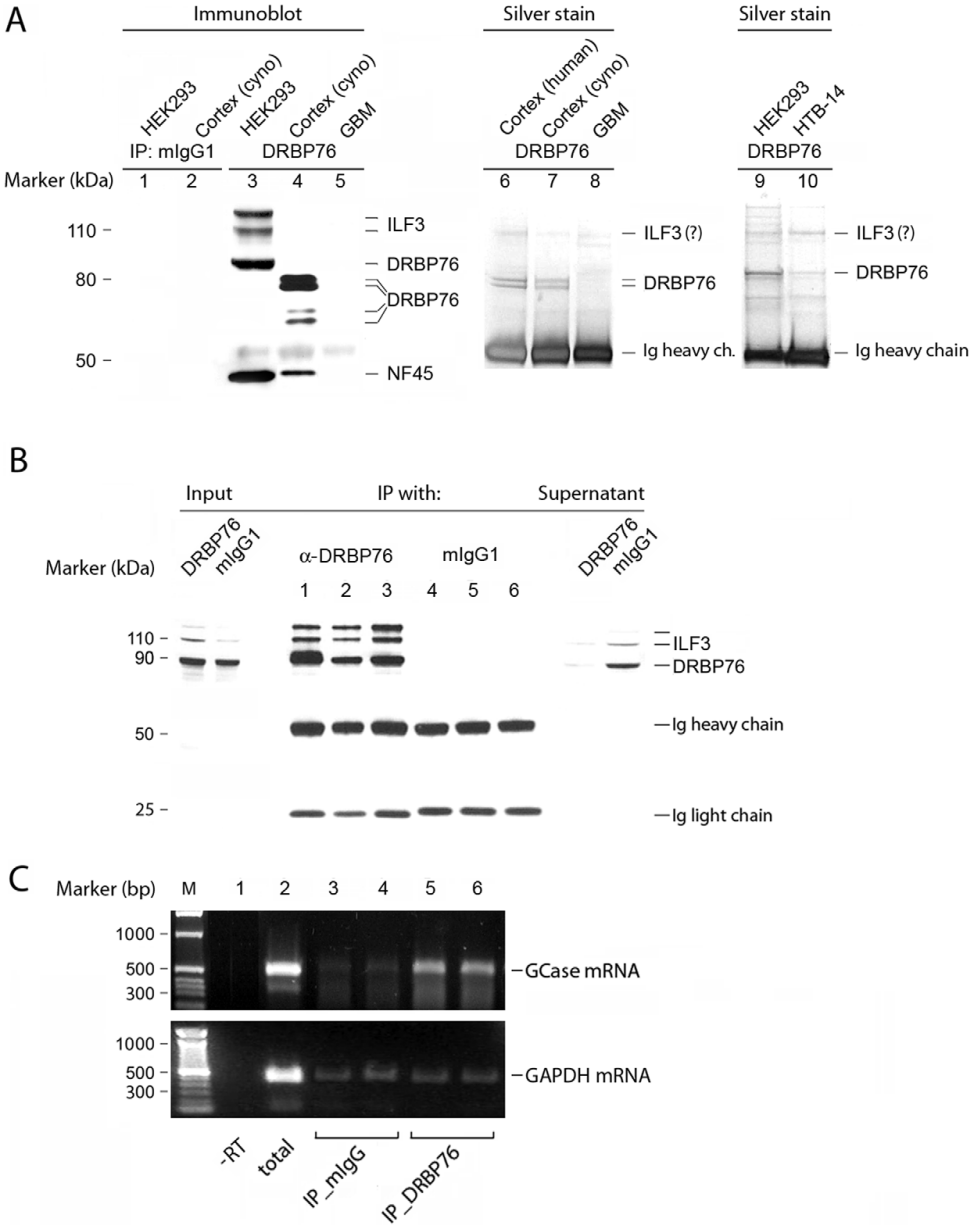
Immunoprecipitation studies of DRBP76. **A**. Left panel. Control mouse IgG and anti-DRBP76 IP from HEK293 cells, normal primate CNS and GBM tissues. Co-IP of NF45 was analyzed as well. Middle/Right panel. Silver stain of anti-DRBP76 immunoprecipitated material from normal human/simian brain and from HEK293 and HTB-14 cell lines, respectively, analyzed by immunoblot. **B**. IP with anti-DRBP76, but not isotype-matched control IgG, effectively removes DRBP76 from HEK293 cell lysates. **C**. RT-PCR of GCase and GAPDH transcript from RNA isolated from immunoprecipitated DRBP76 RNP.

To confirm the identity of DRBP76 immuno-reactive material in cytoplasmic extracts from CNS tissues and to prepare for RIP-Chip analyses of DRBP76, we performed DRBP76 immunoprecipitation (IP) and co-IP analyses of NF45 (Fig. 3A). DRBP76 antibody, but not mouse isotype-matched control IgG, efficiently precipitated the previously observed distinct immunoblot signals from HEK293 cell- and primate brain cytosolic extracts, and yielded co-IP of NF45 in both instances (Fig. 3A, left panel). Silver stain analyses of the DRBP76 IPs from human/macaque normal brain revealed the presence of the characteristic DRBP76 double-band detected in IP immunoblots (Fig. 3A, middle panel). Corroborating the direct immunoblot results (Fig. 1E) and the immunofluorescence data (Fig. 2U–W), DRBP76 IP from cytoplasmic extracts of GBM tissues did not reveal proteins in the ∼75–90 kDa size range (Fig. 3A). We speculate that a faint band at ∼110 kDa in both IPs from normal brain and GBM represents ILF3, since our cytoplasmic extracts typically contain nuclear contaminants (Fig. 1E). Similar IPs from cell lysates produced the expected bands, i.e. the ∼90 kDa and ∼110 kDa NFAR proteins typically isolated from transformed cell lines (Fig. 3A, right panel).

Our observations in CNS tissues, primary CNS tumors and various cell lines raise the possibility that the physiological role of DRBP76 in gene regulation in the CNS may be altered in CNS malignancy. For example, divergent isoform distribution and subcellular localization of DRBP76 is likely to result in distinct association with target mRNAs. To unravel potential roles in gene regulation for DRBP76, we conducted a systematic evaluation of DRBP76's association with potential target mRNAs by RIP-Chip. Ideally, such tests would directly compare mRNA pools associated with DRBP76 isoforms unique to normal human tissues, e.g. brain and transcripts binding to the characteristic ∼90 kDa isoform detected in CNS tumors. We thoroughly evaluated DRBP76 IPs from lysates obtained from monkey and human brain. Due to technical difficulties regarding yield and purity of the IP reactions and the yield and quality of the mRNP co-precipitate, we had to abandon this approach. Since the characteristic DRBP76 isoform distribution in normal tissues is not recapitulated in transformed cells, this left us with performing analyses of the ∼90 kDa form characteristically associated with CNS malignancy or transformed cells. Thus, we carried out RIP-Chip analyses of DRBP76 from HEK293 cells by IP of mRNP complexes and spotted microarray analysis of RNAs associated with them.

First, IP effectiveness and specificity were controlled (Fig. 3). DRBP76 IP efficiently precipitated the protein from HEK293 cytoplasmic extracts, while IP with isotype-matched control mouse IgG neither precipitated DRBP76, nor significantly altered its presence in post-IP supernatants (Fig. 3B). Second, RNA was isolated from the IP reaction and RT-PCR assays were performed by analysis of a known DRBP76 target to evaluate the specificity of the RNP precipitation. Prior studies suggested that cytoplasmic translation control protein (TCP80), a protein with high sequence similarity to DRBP76 and identical dsRNA binding domains [28], binds acid β-glucosidase (GCase) mRNA and inhibits its translation [17]. RT-PCR analysis was performed with total RNA purified from HEK293 cytoplasmic extract, or RNA co-precipitated with DRBP76 antibody or with control mouse IgG. While GCase mRNA was readily amplified from total or positive (DRBP76) IP samples, no significant amplification was observed in mouse isotype-matched IgG controls (Fig. 3C). Amplification of negative control GAPDH mRNA was detected in the total RNA samples, but only trace amounts of signal were obtained from both mouse IgG and anti-DRBP76 IP samples (Fig. 3C).

### 4. Identification of DRBP76-Associated mRNAs

**Figure 4:**
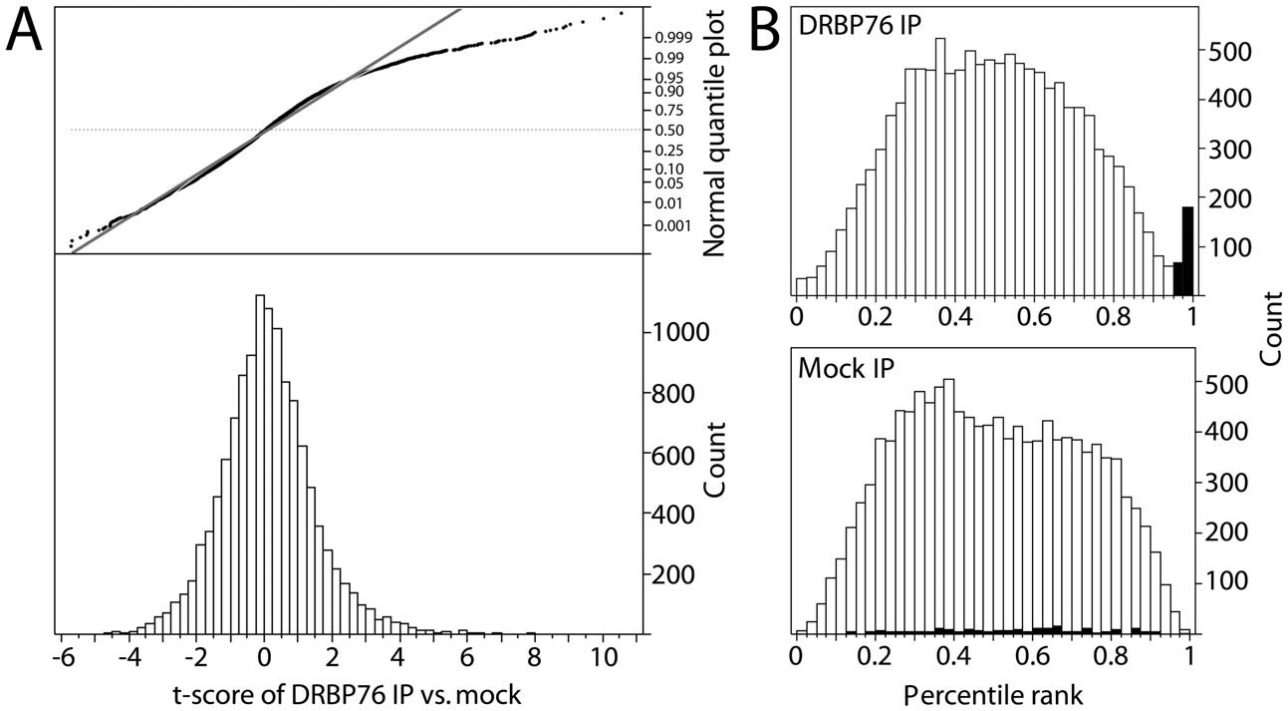
Identification of DRBP76-associated mRNAs using RIP-Chip. **A**. Distribution of DRBP76 IP vs. mock IP t-scores. Deviation of data from the diagonal quantile:quantile plot indicates the non-Gaussian nature of the DRBP76 IP vs. mock IP distribution, corresponding with transcripts specifically enriched in the DRBP76 IP. **B**. Distribution of the average percentile ranks (APR) of 5 biological replicates for DRBP76 IPs and mock IPs. Transcripts considered DRBP76 RNP-associated are indicated by black boxes.

We used RIP-Chip to identify mRNAs associated with RNP complexes containing DRBP76 from cytoplasmic HEK293 extracts. Five biological replicates each of DRBP76, mock (isotype-matched mouse IgG) immunoprecipitates, and total cellular RNA samples were analyzed using spotted cDNA microarrays that interrogated 3.5×10^4^ genes. To qualify for subsequent analysis and be defined as ‘enriched’, a probe had to be >2-fold above local background in any of the IPs or the totals. T-scores, p-values and log-fold changes were calculated comparing DRBP76 IP versus mock IP for all probes expressed (n = 12,468). Visual inspection of the t-score distribution and the quantile:quantile plot indicated the right tail of the DRBP76 vs. mock IP distribution deviated from a normal distribution (Fig. 4A). Since probes with very high t-scores correspond to the right tail, substantially more mRNAs were enriched in the DRBP76 IP than the mock IP.

**Figure 5:**
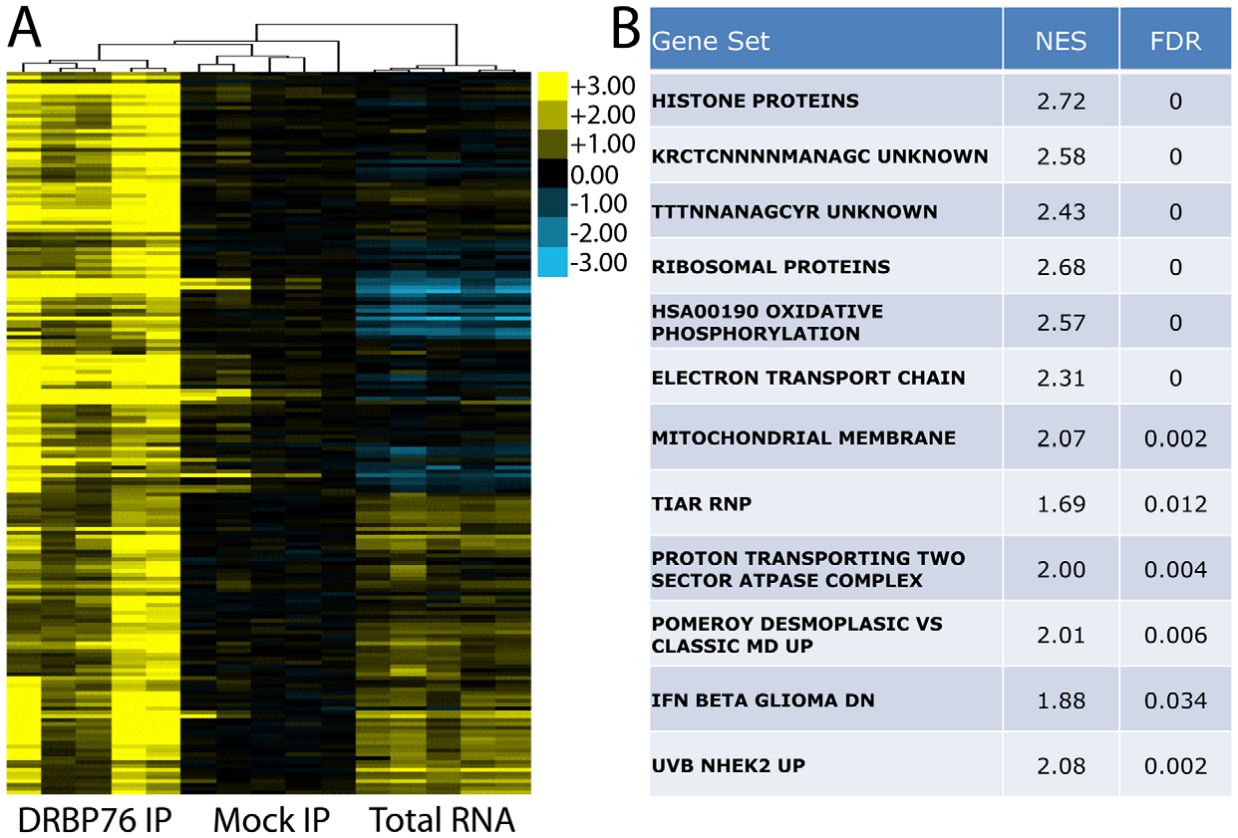
Enrichment of DRBP76-associated mRNAs and their common functional grouping. **A**. Heatmap displaying results of clustering DRBP76 IP, mock IP and total mRNA levels for all DRBP76 targets (n = 189). **B**. Gene sets significantly enriched in the DRBP76 IP vs. mock IP as determined by GSEA. NES =  normalized enrichment score, FDR =  false discovery rate.

To determine a cut-off that may define a discrete population of mRNAs associated with DRBP76, we applied a percentile rank transformation, which was used in the analysis of stem loop binding protein (SLBP) and tris-tetra-proline RIP-Chip targets [29], [30]. The DRBP76 IP average percentile rank (APR) distribution is bimodal at the high percentile ranks, whereas the mock IP APR distribution is normal (Fig. 4B). We defined DRBP76 targets as those with DRBP76 IP APRs >0.95, mock IP APRs <0.95, and a fold enrichment over mock IP >2. Altogether, we detected 189 putative DRBP76 target mRNAs (∼1.5% of the total 12,468 expressed) (Fig. S1). Importantly, for these putative targets relative mRNA abundance did not account for the association of mRNAs with DRBP76 based on the lack of correlation between DRBP76 IPs and totals (r^2^ = 0.04). Further, comparing DRBP76 IPs to mock IPs also exhibited little to no correlation (r^2^ = 0.15), indicating the RIP-Chip successfully isolated a subset of specific target mRNAs. Consequently, DRBP76 IP samples clustered together relative to mock IP samples and total mRNA samples (Fig. 5).

### 5. Common functional groups enriched in DRBP76 IP

**Figure 6:**
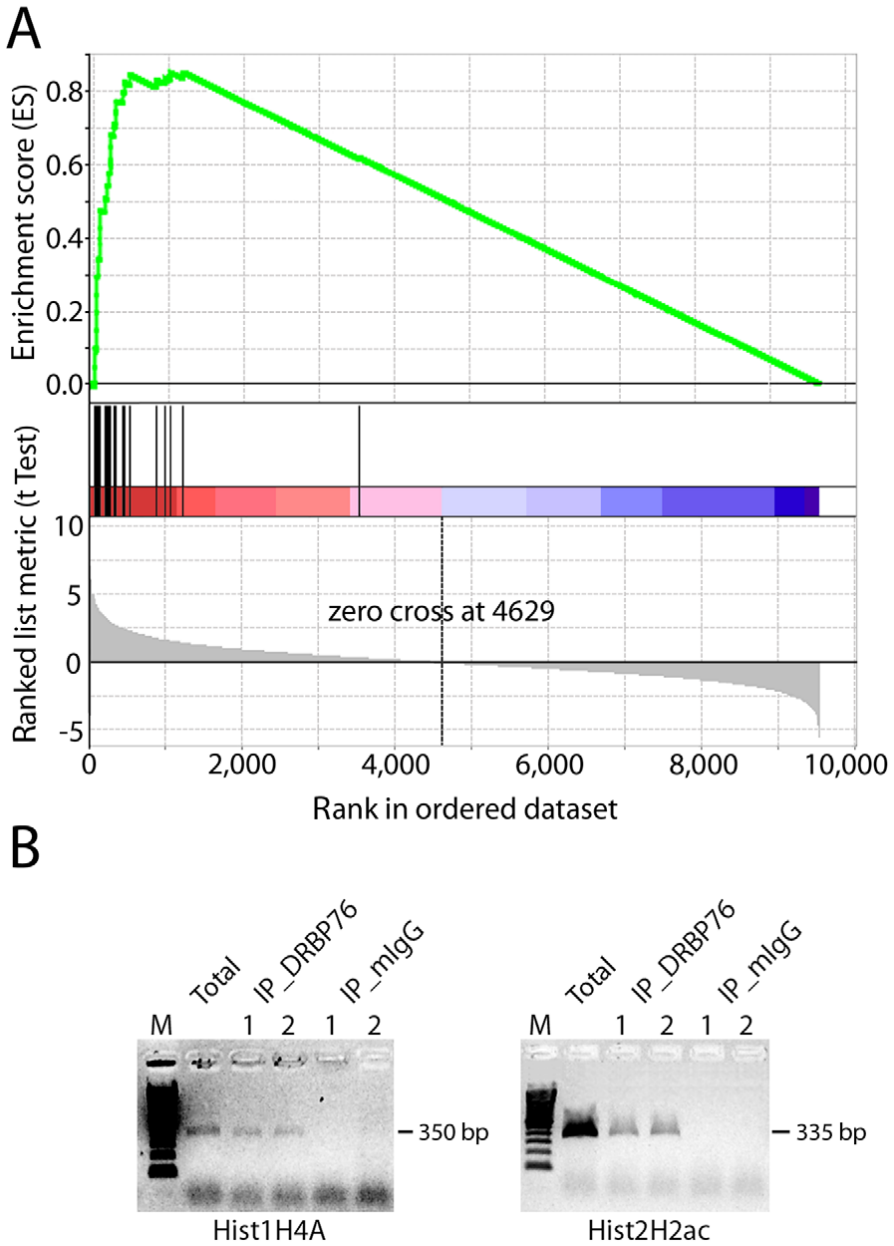
Significant enrichment of DRBP76-associated mRNAs encoding histone proteins in HEK293 cells. **A**. Transcripts rank-ordered by t-scores representing differential enrichment in DRBP76 IP vs. mock IP from HEK 293 cells. Black lines indicate the rank of each individual histone mRNA detected. **B**. Validation of histone mRNA targets by RT-PCR.

We examined the putative DRBP76 target mRNAs, using gene set enrichment analysis (GSEA) to explore known relationships among them and to determine if proteins encoded by these mRNAs are functionally related. GSEA analysis revealed significantly enriched functional categories vital to cellular metabolism (Fig. 5B). These include mRNAs encoding histone proteins, ribosomal proteins, and proteins involved in oxidative phosphorylation. Particularly striking was the enrichment of mRNAs encoding histone proteins. In a list ranked by t-scores representing differential enrichment between of transcripts DRBP76 vs. mock IP, the distribution of histone transcripts was significantly biased for being enriched in the DRBP76 IP (Fig. 6A). Furthermore, gene sets representing mRNAs containing KRCTCNNNNMANAGC or TTTNNANAGCYR motifs were both significantly enriched in the DRBP76 IPs. These motifs are present in histone mRNAs, and overlap with the terminal conserved stem loop (SL) in their 3′UTR [31]. To validate DRBP76 histone mRNA targets, we performed RT-PCR analysis of total RNA isolated from HEK293 cytoplasmic extracts and corresponding RIPs obtained with α-DRBP76 or isotype matched control mIgG antibodies (Fig. 6B). RT-PCR amplified Hist1H4A (∼48.5-fold enriched on microarray) and Hist2H2ac (∼4.9 fold enriched on microarray) from DRBP76 IP, but not from control mouse IgG IP (Fig. 6B).

**Figure 7:**
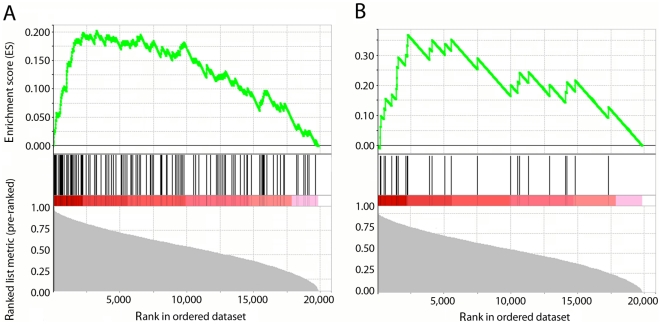
Significant enrichment of DRBP76-associated mRNAs from HEK 293 cells and histone mRNAs in brain tumor derived HTB-14 cells. Transcripts rank-ordered by average percentile rank representing differential enrichment in DRBP76 IP from HTB-14 cells. **A**. Black lines indicate the rank of each individual DRBP76-associated mRNA identified from HEK293 cells (p<0.001). **B**. Black lines indicate the rank of each individual histone mRNA detected (p<0.001).

HTB-14 glioma cells share DRBP76 isoform distribution with HEK293 cells, but the protein is far less abundant in cytoplasm in the former (Fig. 1F), recapitulating the situation in patient GBM tissues (Fig. 1E). To establish whether relative cytoplasmic exclusion of DRBP76 in HTB-14 cells produces distinct association with mRNAs, we conducted RIP-Chip analyses from HTB-14 cell lysates. These revealed that DRBP76-associated mRNAs identified from HEK293 cell were indeed significantly enriched (p<0.001) in DRBP76 RIPs from HTB-14 cells (Fig. 7A). Furthermore, the significant enrichment of mRNAs encoding histone proteins by DRBP76 discovered in HEK293 cells was recapitulated in HTB-14 cells (Fig. 7B). Altogether these data suggest a large degree of similarity between DRBP76 mRNA targets in HEK293 and HTB-14 cells, in line with similar DRBP76 isoform expression in these transformed cell lines.

## Discussion

DRBP76 has been implicated in diverse roles of transcriptional or post-transcriptional gene regulation, e.g., binding the interleukin-2 promoter [32] and stabilizing interleukin-2 mRNA in activated T-cells [15]. Such functional roles are likely influenced by subcellular distribution, which controls binding to target mRNAs or other compartment-specific functions. In tissue culture, DRBP76 is mostly nuclear, but exhibits cytoplasmic re-distribution when prompted by stimuli [21]. Re-distribution may dictate function, either by withdrawing the protein from the nuclear compartment [21] or by enabling interaction with cytoplasmic transcripts [15]. We demonstrated that DRBP76 exhibits intrinsically distinct subcellular distribution in primate tissues in the absence of known stimuli. Mitosis was shown to trigger cytoplasmic re-localization of ∼90 kDa DRBP76 in cultured cells [21]. However, ∼75–80 kDa DRBP76 is present in cytoplasm in mostly post-mitotic brain, while the ∼90 kDa variant is predominantly nuclear in mitotically active GBM.

The distinct electrophoretic mobility of DRBP76 proteins from normal CNS and tumors/cell lines may stem from previously described splice variation affecting exon 3 [25] or the 3′ portion of the DRBP76 transcript [6], [33]. Most likely, splice variation is combined with divergent post-translational modification, e.g. phosphorylation, methylation and acetylation to give rise to heterogeneous DRBP76 proteins in diverse tissues. We are currently using advanced proteomic techniques to elucidate these differences, but preliminary analyses suggest that this is no easy task. It is well known that protein heterogeneity influences subcellular distribution and cellular functions of RNA-binding proteins. A cytoplasmic locale in the CNS may explain DRBP76's association with and axonal transport of Tau mRNA [34] or a role in the replication of neurotropic RNA viruses [18], [19]. However, any *in vitro* study of the true physiologic role of DRBP76 subcellular distribution and its dynamics is limited because the characteristic ∼75–80 kDa DRBP76 isoforms found in normal tissues are absent in tissue culture.

DRBP76 tissue-type specific isoform diversity also has implications for RNA-binding and, thus, post-transcriptional gene regulation. For example, while ∼90 kDa DRBP76 from tissue culture lysates binds efficiently to poly(I):(C) [35], the ∼75–80 kDa isoforms in normal brain do not (Fig. S2). Since the latter do not occur in tissue culture, *in vitro* studies of DRBP76 association with target mRNAs are limited to interpretation of such interactions in cell lines or tumors, where our data suggest a similar ∼90 kDa DRBP76 variant is expressed.

Two previous attempts to identify DRBP76 target mRNAs have been published; both using DRBP76 mRNPs isolated from transformed cells [20], [36]. Formaldehyde crosslinking of epitope-tagged, ectopic protein followed by IP in HEK293 cells yielded a previously unknown class of small, non-coding RNAs, but did not include systematic, genome-wide analyses [36]. Since such non-coding RNAs were not represented on our microarrays, we could not confirm these interactions. We avoided using ectopic epitope-tagged DRBP76 because the protein is abundant in HEK-293 cells and tags can interfere with RNA-binding. Since RIP precipitates existing RNPs, the possibility of blocking RNA recognition due to antibody binding to DRBP76 is remote in our study [22]. Another study reported RIP-Chip of DRBP76 from HeLa cell lysates [20]. The reported target mRNA pool from that study does not overlap with our findings [20]. A number of factors may explain this divergence. In all cell lines tested, the ∼90 kDa DRBP76 variant dominates, but abundance of this protein is inherently divergent in different lines (Fig. 1B). The study by Kuwano et al. used total HeLa cell lysates [20], while our assay employed cytoplasmic extracts. Most importantly, methods to prepare the immunoprecipitated mRNP likely were distinct. We undertook a conscious effort to optimize IP and subsequent DRBP76 mRNP purification steps prior to microarray analysis (Fig. 3). There is little detail regarding these procedures in Kuwano et al. [20], but different experimental protocols may explain divergent outcomes.

The most compelling observation stemming from our studies is the striking cell type-specific isoform distribution of DRBP76 in transformed cells and in primary CNS tumors. Since altered DRBP76 isoform patterns and subcellular distribution were similar in all human tumors and in several cell lines of diverse origin, we speculate that they may be broadly associated with malignancy. The distinct properties of DRBP76 proteins in cancer are likely associated with altered gene-regulatory functions in neoplasia. Our studies indicate strong enrichment of mRNAs encoding histone, ribosomal and oxidative/metabolic functions with the DRBP76 mRNP, consistent with the post-transcriptional operon model of coordinated expression [3]. Cell proliferation depends upon coordinated histone production and protein biosynthetic machinery that principally involves ribosomal proteins [30], [37]–[40]. Moreover, posttranscriptional coordination of oxidative pathways and mitochondrial functions are well documented in *S. cerevisiae* [41]–[43], yet this has not been elucidated in mammalian systems. Whether DRBP76 serves such functional roles by association with mRNAs encoding these functionally related mRNAs is yet to be determined. In any case, there is abundant literature demonstrating the importance of ribosomal biosynthesis and oxidative metabolism involving mitochondria and the Warburg effect in many pathogenic processes including carcinogenesis [1], [44], [45]. It will be important to determine whether DRBP76 participates in coordinating these overlapping functions and the extent to which they are altered in malignancy.

## Materials and Methods

### Ethics statement

All human CNS and CNS tumor tissues used in this study were existing, discarded surplus pathological specimens. No human subjects were enrolled for research purposes and this study was declared exempt from review by the Institutional Review Board (IRB).

Vertebrate animal tissues (from mouse and *Cynomolgus* macaque) were existing, discarded surplus materials stemming from unrelated studies. No animals were enrolled in research activities for purposes related to this study and the tissues were not collected with the purpose of being used in this study. Therefore, the use of murine and simian tissues in this study does not qualify as vertebrate animal research. This was confirmed by the Director of the Institutional Animal Care and Use Committee (IACUC) at Duke University Medical Center.

### Cell lines, preparation of cytoplasmic extracts and immunoblot

HEK293, HTB-14, and SH-SY5Y cells (ATCC, Manassas, VA) were grown in Dulbecco's Modified Eagle Medium (DMEM) supplemented with 10% FBS, nonessential amino acids and antibiotics. NHA (Lonza, Walkersville, MD) were propagated following the manufacturer's suggestions. Cells were scraped from culture dishes, washed with cold PBS and pelleted by centrifugation at 4°C. The pellet was re-suspended in an equal volume of polysome lysis buffer (PLB; 100 mM KCl, 5 mM MgCl_2_, 10 mM HEPES-KOH, pH 7.0, 0.5% NP-40, 1 mM DTT and 1 mM PMSF) supplemented with RNaseOUT (Invitrogen, Carlsbad, CA), protease inhibitor cocktail (Sigma, St. Louis, MO) and Halt™phosphatase inhibitor (Pierce, Rockford, IL). Lysates were incubated on ice for 5–10 min and stored in aliquots at −80°C. The total protein concentration of cytoplasmic lysate determined by Bradford assay was 7–10 mg/mL. Cell lysates were fractionated using NE-PER Nuclear and Cytoplasmic Extraction Reagents (Pierce, Rockford, IL) following the manufacturer's instructions. Induction of a neuron-like phenotype in SH-SY5Y cells with ATRA was carried out as reported before [46]. Normal simian brain tissues were obtained from the Center of Biological Evaluation and Research, Food and Drug Administration and mouse tissues were surplus discarded pathological specimens stemming from unrelated studies in the laboratory. It is estimated that the autolysis time for fresh frozen monkey and mouse tissues was less then 30 min. Normal human brain tissues were obtained from the New York Brain Bank, Columbia University. The tissues were dissected during autopsy from deceased patients without history of neurological illness. The approximate autolysis time for human brain samples was 24–32 hours. Human glioblastoma multiforme (GBM) samples were fresh-frozen, surplus pathological specimens obtained at the time of craniotomy. Tissue samples were gently thawed, dissected into ∼1 mm^3^ fragments, washed with cold PBS and homogenized in 5 volumes of cold brain lysis buffer (BLB; 150 mM NaCl, 50 mM HEPES-KOH, pH 7.3, 10% glycerol, 1 mM EDTA, 5 mM EGTA, 0.5% NP-40, 2.5 mM DTT and 1 mM PMSF) supplemented with RNAse, phosphatase and protease inhibitors as described above. The samples were lysed on ice for 30 min and stored in aliquots at −80°C. Thereafter, lysates were centrifuged and supernatants purified with Handee™ Spin Columns (Pierce). Pellets were re-suspended in equal volume of lysis buffer and used as nuclear fraction. Total protein concentration in cytoplasmic lysates was determined by Bradford assay. For immunoblot analyses, proteins were resolved by electrophoresis in 4–12% Bis-Tris NuPAGE gels (Invitrogen) and transferred to PROTRAN nitrocellulose membrane (Schleicher & Schuell, Keene, NH). Seven % Tris-Acetate NuPAGE gels (Invitrogen) were used for more efficient protein separation in 70–100 kDa region. After overnight blocking in Startingblock T20 buffer (Pierce) membranes were incubated with anti-DRBP76 antibody (BD Biosciences, Franklin Lakes, NJ) for 1 h, followed by three washes with PBST (1xPBS, 0.1% Tween-20) and 1 h incubation with secondary HRP-conjugated anti-mouse antibody. After three washes, signal was detected by enhanced chemiluminescence (West Pico Substrate; Pierce) followed by autoradiography.

### Immunofluorescence

Normal human brain tissues were surplus pathological specimens (parietal cortex) obtained during craniotomy from epilepsy patients histopathologically confirmed to consist of normal human CNS. GBM patient samples were histopathologically confirmed to be free of contaminating CNS. Immunostaining of normal human brain and GBM cryosections (4 µm) was performed as follows. Sections were fixed with cold 4% paraformaldehyde in PBS for 15 min, permeabilized with −20°C methanol for 10 min, blocked with 5% goat serum in PBS for 1 h and incubated overnight at 4°C with anti-DRBP76 antibody diluted in blocking buffer at 1∶1000 or equal amount of isotype-matched mouse IgG1 control to rule out nonspecific staining. Then, slides were washed with PBS and incubated for 45 min at room temperature with FITC-conjugated anti-mouse IgG (Sigma) at a 1∶150 dilution. After 3 washes with PBS, normal brain sections were treated with NeuroTrace Fluorescent Nissl Stain (Invitrogen), a neuronal cell marker. Staining was performed for 20 min at room temperature with red NeuroTrace stain at a 1∶1000 dilution. Following 2 h wash with PBS, both normal brain and GBM sections were treated with 1 mM CuSO_4_ in 50 mM NH_4_OAc (pH 5.0) for 90 min at room temperature to reduce lipofuscin autofluorescence [47]. Sections were washed and mounted in ProLong Gold antifade reagent with 4′,6′-diamidino-2-phenylindole (DAPI; Invitrogen). Images were collected using an XI50 Olympus microscope, DP70 digital camera, and DPController/DPManager software. Images were processed and analyzed using Adobe Photoshop software.

### IP of endogenous DRBP76 RNP complexes

IP of mRNP complexes from HEK293 or HTB-14 cytoplasmic lysates was generally carried out as described before [22]. Briefly, protein-G sepharose beads were pre-swollen in NT2 buffer (150 mM NaCl, 50 mM tris-HCl, pH 7.5, 1 mM MgCl_2_, 0.05% NP-40) supplemented with 1% BSA and coated with 20 µg of DRBP76 antibody or mouse IgG1 as isotype negative control (both BD Biosciences) overnight at 4°C. Unbound antibody was rinsed off 5 times with 1 mL of cold NT2 buffer and beads were incubated with pre-cleared cytoplasmic lysate for 4 hrs at 4°C. Thereafter, supernatant was collected for immunoblot/RNA isolation and beads were washed twice with BD buffer (150 mM NaCl, 10 mM tris-HCl, pH 7.5, 0.5% NP-40, 1% Triton X-100, 1 mM EDTA, 1 mM EGTA and 1 mM DTT) supplemented with 1 M urea and 4 times with BD buffer only. During the last wash 10% of beads were removed for immunoblot and the remaining beads were resuspended in 100 µL NT2 buffer and processed for RNA isolation. The amount of cytoplasmic lysate used for each IP was equivalent to 1 mg of total protein.

### RNA purification and reverse transcription (RT)-PCR

RNA bound to the beads after IP with DRBP76 antibody or mouse IgG1 control, or total RNA from cytoplasmic lysates was purified using Trizol Reagent (Invitrogen) as described elsewhere [22]. The RNA concentration and purity (OD260∶280 ratio) was determined on a Nanodrop spectrometer and the integrity of the RNA samples was verified by electrophoresis using the Reliant Gel System (Cambrex, East Rutherford, NJ). Ten µg of RNA per sample were submitted to the Duke University Microarray Core Facility for spotted array analysis. Five biological replicates of each sample were analyzed. RNA from total lysate and from the IPs was subjected to RT with oligo-dT (Ambion, Foster City, CA). Gene specific primers and Sahara Mix (Bioline, Taunton, MA) were used to amplify target genes identified in microarray. These tests included primer pairs (1) 5′-actcaccacaatgtccgcct-3′ and (2) 5′-ggagccctcaggaatgaact-3′ (GCase), (3) 5′-gtctggacgtggtaaggg-3′ and (4) 5′-gcctttggttcagaaatgcaag-3′ (Hist1 H4A), (5) 5′-ctggtcgtggcaaacaag-3′ and (6) 5′-cctggatgttaggcaaaacg-3′ (Hist2 H2ac) or (7) 5′-catgttcgtcatgggtgtgaacca-3′ and (8) 5′-agtgatggcatggactgtggtcat-3′ (GAPDH).

### Microarray data analysis

Arrays were printed at the Duke Microarray Facility using the Genomics Solutions OmniGrid300 Arrayer and contained Human Operon v4.0.1 oligo set (Oligo Source) consisting of ∼3.6×10^4^ unique 70-mers. For all arrays RNA was assayed using direct labeling of experimental samples (Cy 3) and Stratagene Universal Human Reference RNA (Cy 5). Array data were submitted to the GEO, GSE20001. All arrays were subject to loss normalization within each array and scale normalization across arrays using Array Magic [48]. Replicate probes were collapsed to the median value. Probes had to have signal >2-fold than local background in all biological replicates for any of the RIPs or the totals at any time point to be considered for subsequent analysis. Gene Pattern [49] was used to calculate t-scores, p-values and log fold changes comparing the DRBP76 and mock IPs. Gene set enrichment analysis (GSEA) [50] was used to determine gene sets significantly enriched in the DRBP76 IP. Only gene sets with false-discovery-rate (FDR) q-values of<0.05 or family-wise-error-rates (FWER) of<0.1 were considered significant. For each probe, a DRBP76 IP average percentile rank and mock IP average percentile rank was calculated as the average of the individual percentile ranks from each of the 5 biological replicates of the DRBP76 and mock IPs, respectively. Probes with a DRBP76 IP average percentile rank (APR) of >0.95, mock IP APR of<0.95, and a fold enrichment over mock IP>2 were considered DRBP76 targets. A heatmap depicting their relative hybridization values in the DRBP76 IP, IgG IP and total mRNA was created using Gene Pattern and Java Treeview [51].

## Supporting Information

Figure S1 List of putative DRBP76 target mRNAs in HEK293 cells.
(0.16 MB XLS)

Figure S2 Poly(I):(C) binding studies. Binding of DRBP76 proteins from HEK293 cells (top) and Cynomolgus brain lysates (bottom) to poly(I):(C) sepharose.
(0.59 MB TIF)
