# Supplementary figures and images for: Tissue Type-Specific Expression of the dsRNA-Binding Protein 76 and Genome-Wide Elucidation of Its Target mRNAs
Source: PLoS One. 2010 Jul 23;5(7):e11710. doi: 10.1371/journal.pone.0011710 (PMC2909144; doi:10.1371/journal.pone.0011710)

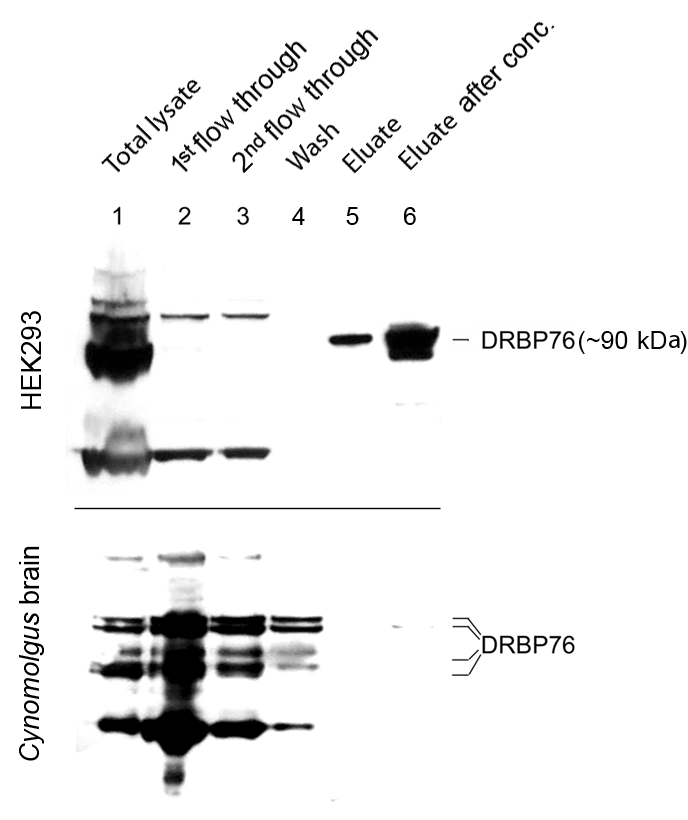

Supplement: Figure S2 — Poly(I):(C) binding studies. Binding of DRBP76 proteins from HEK293 cells (top) and Cynomolgus brain lysates (bottom) to poly(I):(C) sepharose. (0.59 MB TIF) [file pone.0011710.s002.tif]
